# Eyeglasses Lens Contour Extraction from Facial Images Using an Efficient Shape Description

**DOI:** 10.3390/s131013638

**Published:** 2013-10-10

**Authors:** Diana Borza, Adrian Sergiu Darabant, Radu Danescu

**Affiliations:** 1 Computer Science Department, Technical University of Cluj Napoca, 28 Memorandumului, Cluj Napoca 400114, Romania; E-Mail: borza_diana@yahoo.com; 2 Computer Science Department, Babes Bolyai University, 58-60 Teodor Mihali, C333, Cluj Napoca 400591, Romania; E-Mail: adrian.darabant@tvarita.ro

**Keywords:** eyeglasses detection, *Fourier descriptors*, Monte Carlo methods

## Abstract

This paper presents a system that automatically extracts the position of the eyeglasses and the accurate shape and size of the frame lenses in facial images. The novelty brought by this paper consists in three key contributions. The first one is an original model for representing the shape of the eyeglasses lens, using *Fourier descriptors*. The second one is a method for generating the search space starting from a finite, relatively small number of representative lens shapes based on Fourier morphing. Finally, we propose an accurate lens contour extraction algorithm using a multi-stage Monte Carlo sampling technique. Multiple experiments demonstrate the effectiveness of our approach.

## 1. Introduction

Modern eyeglasses lens manufacturing requires a complex process, called centering, that involves the measurement of several morphological parameters of the patient's face and their correlation with morphological parameters of the frames that the patient will wear. The most important morphological parameters regarding the eyeglasses are: the frame *bridge* (the distance between the left and right rims of the eyeglasses), the *boxing* size (height and width) for each lens, the *fitting height* (the distance between the bottom of the lens and the eye pupil) and the *vertex distance* (the distance between the interior surface of the lenses and the cornea).

The classical tools and methods for measuring these morphological parameters usually involve manual processing; therefore, they are error prone and do not provide the required accuracy. Recently, most of the large eyeglasses producers ([1,2]) developed computer-aided systems that can accurately measure several morphological parameters by capturing and processing a digital image of the patient.

An eyeglasses detection algorithm can be used to automatically detect the key features needed by these systems in the measurement process. For example, the bridge, the boxing and the fitting height can be easily measured with the use of a computer vision system if the eyeglasses and the lens contours are accurately detected and extracted from the input image.

Eyeglasses are one the most important factors that affect the performance of face recognition algorithms, which are crucial in a variety of applications, such as security and surveillance systems and human computer interaction. The robustness of the facial recognition systems can be increased if the eyeglasses are accurately detected and removed from the subject's face.

Finally, from an esthetic perspective, the eyeglasses rim contours can be fed to an augmented reality system that would allow a person to observe his or her appearance with different colors of the eyeglasses rims or even without glasses (if eyeglasses removal is performed). In addition, if the contour of the eyeglasses is accurately determined, its shape can be modified, so that the patient can customize the shape of the rims.

We propose a novel approach for accurately determining the position of the eyeglasses and the exact contour and size of the lenses based on a multistage Monte Carlo method. The first step of the algorithm is to determine the approximate region of the eyes; based on the position of the eyes, the eyeglasses search region is determined to be similar to the method presented in [3]. Next, we use a Monte Carlo search method to determine the approximate position and size of the eyeglasses. The search space is explored by morphing between a finite set of key hypotheses stored in a database. In the final step of the algorithm, a random walk is performed around the previously determined solution in order to finely tune the position, size and shape of the eyeglasses. The algorithm is mainly designed for optometric measuring devices, such as [1,2,4], which process one or multiple high resolution facial images of the patient in order to compute several morphological parameters needed by eyeglasses prescriptions.

This work will highlight the following contributions:

- An original method for accurately detecting the position of the eyeglasses and the accurate shape and size of the eyeglasses lenses;
- An original eyeglasses model: the shape of the eyeglasses rims is described using *Fourier descriptors*; their size is controlled by a single parameter, namely, the interpupillary distance, while their position is determined by the localization of the eyes. This model can be used both for detection and tracking of the eyeglasses rims;
- An original method for morphing between shapes using *Fourier descriptors* . As the input domain is large, we only use a small set of representative shapes and dynamically generate the search space by morphing elements of this set;
- A lenses contour extraction method based on a multistage Monte Carlo search, using the proposed model.

The remainder of this paper is organized as follows. Section 2 presents a general review of the most relevant research literature on eyes and eyeglasses detection, localization and removal. In Section 3, we present the outline of the proposed solution, and in Section 4, we detail this solution. Experimental results on eye detection and eyeglasses rim extraction are presented in Section 5. In Section 6, we perform a comparative study between the previous methods presented in the literature and our eyeglasses extraction algorithm. We conclude this paper in Section 7.

## 2. Related Work

Eyeglasses detection was pioneered by Jiang *et al.* [3], who proposed six measures for determining the presence of eyeglasses in facial images based on edge information from two regions around the eyes. This method requires that the eyes are detected in advance and does not provide any information regarding the localization or the shape of the eyeglasses.

Jing *et al.* [5] extended the methods for detecting the presence of eyeglasses presented in [3], and extracted the eyeglasses based on deformable contours, using geometrical and edge features. The final position of the eyeglasses is determined by means of dynamic programming. This method is based only on edge information and gives inaccurate results, because of the presence of eyebrows, wrinkles, and so on.

To overcome this limitation, Jing *et al.* [6] proposed a method for eyeglasses detection and extraction using Bayes' rule. Prior to glasses detection, a supervised learning scheme is used to learn the visual features of the glasses. Feature vectors are extracted for each edge point in the test image, and a Bayesian classifier is used to determine the points that belong to the glasses class. This method has a better performance than the work presented in [5], but requires knowledge about the eye position, and the faces in the test images must be normalized.

Park *et al.* ([7,8]) presented a method for eyeglasses removal in facial images based on PCA (*Principal Component Analysis*) reconstruction. The eyeglasses points are extracted from a color input image using the Generalized Skin Color Distribution ([9,10]) transformation and edge information. The facial image without glasses is determined by example-based learning, and finally, the image is updated by means of recursive error compensation using PCA reconstruction.

Wu *et al.* ([11]) proposed a method for eyeglasses detection that does not require any information regarding the position of the eyes, the face pose or the shape of the eyeglasses. The proposed method uses a trinocular vision system and makes use of the 3D Hough Transform in order to determine the 3D plane passing through the rims of the eyeglasses. The eyeglasses rims are extracted based on the determined 3D plane and additional geometrical constraints. This method requires a more complex vision system and has a greater computational complexity than 2D methods.

More recently, Wu *et al.* ([12]) presented a system for automatically removing eyeglasses in facial images. First, the approximate region of the eyes is detected, and then, a Markov Chain Monte Carlo method is used to determine 15 key points on the eyeglasses by searching for the global minimum of the *a posteriori* probability distribution. Finally, the eyeglasses are removed from the input image using statistical methods to learn the mapping between pairs of face images with and without eyeglasses from a database.

Xiao *et al.* [13] proposed a method based on Delaunay triangulation to detect the eyeglasses in frontal facial images. A Delaunay triangulation is performed on the binarized input image, and dense points in the facial region are clustered by measuring Delaunay triangles of different sizes. The eye region corresponds to small-sized triangles and triangle chains with a short length and narrow width. Eyeglasses are immediate neighbors of the eyes and are detected by specific Delaunay triangles surrounding the eye regions.

Xiaodong *et al.* [14] proposed an eyeglasses detection and removal algorithm, which is not sensitive to illumination and contrast variations, based on phase congruency and progressive inpainting techniques.

In [15], Zheng presents a method for deciding whether the eyeglasses are present or not in a thermal infrared image. The method is based on the fact that glasses appear as dark areas in a thermal image. Using this property, the eyeglasses are detected based on geometrical conditions (area, shape and location of the eyeglasses).

The lens extraction algorithm we propose requires that the location of the eyes is estimated within a certain error margin in the facial image. In the past several years, active research and significant progress has been made in the area of eye and facial feature detection in general. For example, in [16], the authors present a detailed survey of the recent eye detection techniques, emphasizing the challenges they pose, as well as the importance of eye detection in many problems in computer vision and other areas.

The work presented in this paper aims at extending the functionality and performance of the state-of-the-art systems, by estimating the exact position of the eyeglasses rims, as well as the exact contour and size of the lenses.

## 3. Solution Outline

**Figure 1.**
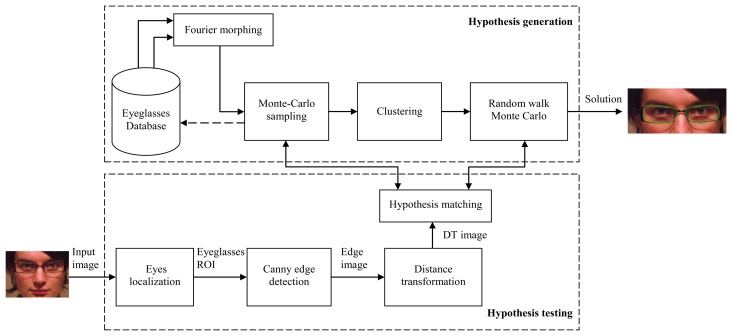
Eyeglasses detection algorithm outline.

Our approach for detecting the eyeglasses is based on hypothesis testing. We created a database that contains different key shapes of eyeglasses rims, represented by *Fourier descriptors*. The eyeglasses search space is explored by performing linear morphing between the Fourier descriptors of key models stored in the database. These key models are grouped into classes based on their shape similarity. Morphing is only performed between elements belonging to the same class in order to obtain realistic eyeglasses shapes. The algorithm presented in Figure 1 is applied for each of one of these classes.

The eyeglasses detection algorithm requires that the position of the eyes is known in the input image. Therefore, the first step of the algorithm is to detect the position of the eyes and to establish the eyeglasses region of interest (ROI), based on the eye position.

Next, a multistage hypotheses testing method is used to determine the position of the eyeglasses and the exact contour and size of the lenses. In the first stage, a Monte Carlo search is performed. A large number of hypotheses are generated and matched against the input image in order to determine the extent to which they fit in the image. Then, the hypotheses are grouped into clusters based on their position and size. The best hypothesis from the cluster with the highest matching score is chosen as the initial sample for the second stage: random walk Monte Carlo, which explores the search space in a region that surrounds the initial solution, in order to determine the rims' final shape, size and position.

## 4. Solution Description

### 4.1. Shape Description

The eyeglasses rims come in very different shapes; therefore, a parametric description of their contour proves to be very difficult. In order to represent the rim shapes, Fourier descriptors are used [17].

The Fourier descriptors are computed by taking the Fourier transform of a shape, where every point on the contour (*x*, *y*) is mapped to a complex number *z*(*i*) = *x*(*i*)+*jy*(*i*), where the *x*-axis is considered the real axis and the *y*-axis is considered the imaginary axis:

(1) $$c(k)=\overset{N-1}{\underset{i=0}{\text{∑}}}z(i)e^{-j2\pi ki/N}$$

The complex coefficients, *c*(*k*), are called the shape's *Fourier descriptors*. The initial shape can be restored by applying the inverse Fourier transform on these descriptors:

(2) $$z(i)=\frac{1}{N}\overset{N-1}{\underset{k=0}{\text{∑}}}c(k)e^{-j2\pi ki/N}$$

This representation describes the frequency information of the shape: lower coefficients provide information about the rough shape, while higher order coefficients account for shape details.

Fourier descriptors can be easily interpreted. The first coefficient, *c*(0), represents the centroid of the shape and is the only coefficient that encodes information about the shape position. The second frequency component, *c*(1), describes the size of the shape; eliminating all but the first two *Fourier descriptors*, the reconstructed shape will always result in a circle (an *n*-sided polygon).

**Figure 2.**
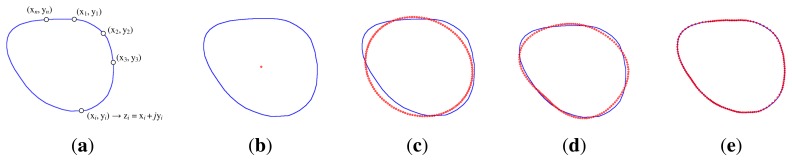
Reconstruction of the rim contour using *Fourier descriptors*. (**a**) Initial shape; (**b**) one descriptor; (**c**) two descriptors; (**d**) six descriptors; (**e**) 14 descriptors.

When reconstructing the shape, if only a few lower frequency coefficients are chosen, then an approximate outline of the initial shape will be obtained, while if higher frequency descriptors are used, then the shape will be described in more detail. The same number of points exists in the shape's representation, but less information is used for representing each point [18]. Figure 2 shows the boundary of an eyeglasses rim reconstructed based on increasing the number of *Fourier descriptors*.

The main reason for the popularity of the Fourier descriptors is their invariance to common geometric transformations, such as translation, scaling and rotation.

In order to translate a shape with a point Δ*_xy_* = Δ*x* + *j*Δ*y*, this point must be added to each point on the shape contour: *z_t_*(*i*) = [*x*(*i*) + Δ*x*] + *j*[*y*(*i*) + Δ*y*]. All information regarding the shape position is contained in the first descriptor of the shape *c*(0), so translation only affects this descriptor: *c_t_*(0) = *c*(0) + Δ*_xy_*. Translation-invariant Fourier descriptors are obtained by leaving out the first descriptor: *c*(0).

If the contour of a shape is scaled with a coefficient, *α*, *z_s_*(*i*) = *αz*(*i*), the magnitudes of the coefficients are also scaled with this coefficient: *c_s_*(*k*) = *αc*(*k*). Scaling invariance is achieved by normalizing all *Fourier descriptors* by |*c*(1)|.

**Figure 3.**
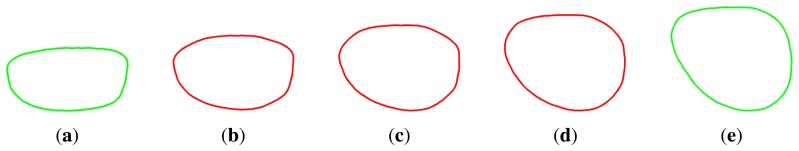
Morphing between two shapes using *Fourier descriptors*. (**a**) Shape 1, *β* = 0; (**b**) *β* = 0.25; (**c**) *β* = 0.5; (**d**) *β* = 0.75; (**e**) Shape 2, *β* = 1.

In order to generate a new shape out of two existing shapes, we make use of the closure property of Fourier series: the sum of any two Fourier series is itself a Fourier series. This means that we can obtain an intermediary shape between any two given shapes by using a linear combination of the boundaries of the two initial shapes:

(3) $$C_{m}=\beta C_{s1}+(1-\beta )C_{s2},\;\beta \in [0,1]$$

where *C_s_*_1_ represents the Fourier descriptors of the first shape, *C_s_*_2_ represents the Fourier descriptors of the second shape and *β* is the morphing factor. Figure 3 shows the result of morphing between two shapes with different morphing factors.

We heuristically determined that the number of *Fourier coefficients* necessary to describe the rims boundaries is 14. The main advantage of this shape representation is that with a limited number of variables (14 *Fourier descriptors*), we were able to accurately describe a variety of eyeglasses rims.

The following parameters are sufficient for describing a hypothetical eyeglasses rim:

(4) $$v=\left[\begin{array}{c} \text{class}-\text{shape class}\\ \left(x,y)-\text{shape position}(\text{the centroid}\right)\\ \text{size}-\text{shape size}\\ \{S_{1},S_{2}\}-\text{reference shapes}\\ \beta -\text{interpolation factor} \end{array}\right]$$

The shape of a hypothesis is determined by the two shapes, {*S*_1_, *S*_2_}, selected from the database, which are morphed with the morphing factor, *β*. The shapes {*S*_1_, *S*_2_} belong to the same class, *class*, and are represented by Fourier descriptors. The shape and position of the hypothesis are controlled by the *size* attribute and the shape's centroid coordinates (*x*, *y*), respectively.

All the geometrical transformations (scaling, translation, flipping, morphing) are performed in the Fourier space.

### 4.2. Hypotheses Validation and Matching

In order to estimate the extent to which the generated hypothesis matches the test image, we perform an edge detection [19], followed by a distance transformation (DT) [20]. The boundary of the hypothetical shape is then superimposed onto the DT image, and the matching score of the hypothesis is computed as the average of all the pixels from the distance transform image that lie under its boundary.

(5) $$\text{mathing}\_\text{score}(\vartheta )=\frac{1}{\left|\vartheta \right|}\sum_{(x,y)\in \vartheta }DT(x,y)$$

where *ϑ* represents the contour of the shape.

The final matching score of a hypothesis is computed as the average between the matching score of the left rim and the matching score of the right rim.

(6) $$\text{score}(\text{hypothesis})=\frac{\vartheta_{1}+\vartheta_{2}}{2}$$

where *ϑ*_1_ and *ϑ*_1_ represent the contour of the left eyeglasses rim and right eyeglasses rim, respectively.

**Figure 4.**
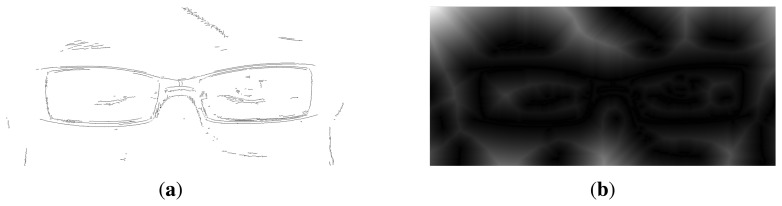
Edge and Distance transform images. (**a**) Canny edge detection; (**b**) distance transform.

Figure 4 depicts the Canny edge and the corresponding distance transformation. Here, the edge image is close to the ideal case, where the shape and the position of the lenses seem easy to extract. However, in the majority of cases, the contours of the lenses have large gaps and many occlusions from other elements, such as eyebrows, light reflection, and so on.

In addition, a hypothesis must comply with several geometrical conditions in order to be considered a potential solution. These conditions regard:

- *The position* of the rims: the eyes must be enclosed by the rims, and the eyeglasses centroid must be close to the eyes center;
- *The size* of the rims: the size of the rims must be larger than the size of the eyes.

The neoclassical canon for facial proportions ([21]) divides the face vertically into five equal parts, assuming that the intercanthal distance (which occupies the middle fifth) is equal to the nasal width and widths of the eyes. We used this relation to further restrain the size and the region in which the eyeglasses rims can be localized.

**Figure 5.**
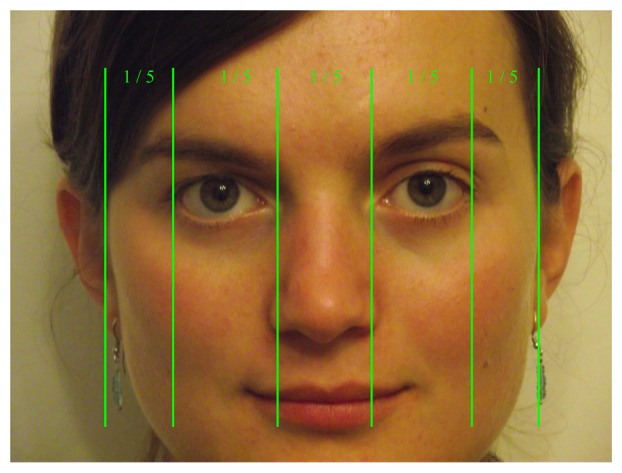
Neoclassical cannon (inspired by [21]). Vertical fifths of the human face; the eye usually measures one fifth of the human face.

The eyeglasses' vertical sides must lie in the first and middle fifth region of the face for the left lens and in the middle and fifth region for the right lens, as depicted in Figure 5.

### 4.3. Eyeglasses Database

For the generation of a new hypothesis shape, we use a database that contains the Fourier descriptors of the left eyeglasses rims; the corresponding right rim is automatically generated by horizontally flipping the left rim. In this way, the symmetry between the left and the right rim is implicitly contained in the model.

To create the database, the boundaries of different eyeglasses rims were manually selected from several facial images. Next, each boundary was described using *Fourier descriptors*, which were normalized to scaling and translation. The database contains 40 samples of eyeglasses rims. The search space of the eyeglasses shapes is covered by morphing between the shapes contained in this database.

Eyeglasses rims are grouped into three different classes based on their shape similarity: symmetrical rectangular rims, elliptic symmetrical rims and asymmetrical rims. Table 1 presents samples from each one of the eyeglasses class: the contour of the eyeglasses rim, as well as the *Fourier descriptors* of the contour. In our experiments, we used 14 *Fourier descriptors* to describe the contour of the eyeglasses rims, as previously stated in Section 4.1.

Each time a new hypothesis is generated, two random shapes (belonging to the same class) are selected from this database and morphed with a randomly selected factor.

### 4.4. Eyes Localization

For determining the approximate position of the eyes, we used the well-known Viola-Jones [22] framework, as it provides high accuracy in real time. The algorithm is based on several key features: the use of simple rectangular features, called *Haar*-*like features*, a novel image representation technique, *Integral image representation*, which allows the features to be computed very quickly, the AdaBoost algorithm [23] and a method for combining increasingly complex classifiers into a *cascade*, which rapidly discards the background pixels. Although this algorithm was initially proposed for face detection, it can be trained for any object. We used the training data provided by the OpenCV framework for the eye detection.

The first step of the eye detection algorithm is to detect all the candidate regions for the eyes (separately for the left and for the right eye) using the Viola-Jones algorithm. Next, all the regions that do not have a left eye-right eye pair are discarded, and the candidate regions are split into left and right groups with respect to the center of all the candidate regions. After this step, all the eye zones that overlap are merged into their enclosing rectangle. Finally, the best matching group and the localization of the eyes are computed based on the the number of candidate eye zones contained and the dimension of the enclosing rectangle. The center of each eye is determined by computing the centroid of the overlapping left (or right) eye zones, and the area of the eye is computed from the sequence of Haar-detected eye zones, as a value between the size of the smallest eyes zone and the mean of all the detected eye zones.

**Figure 6.**
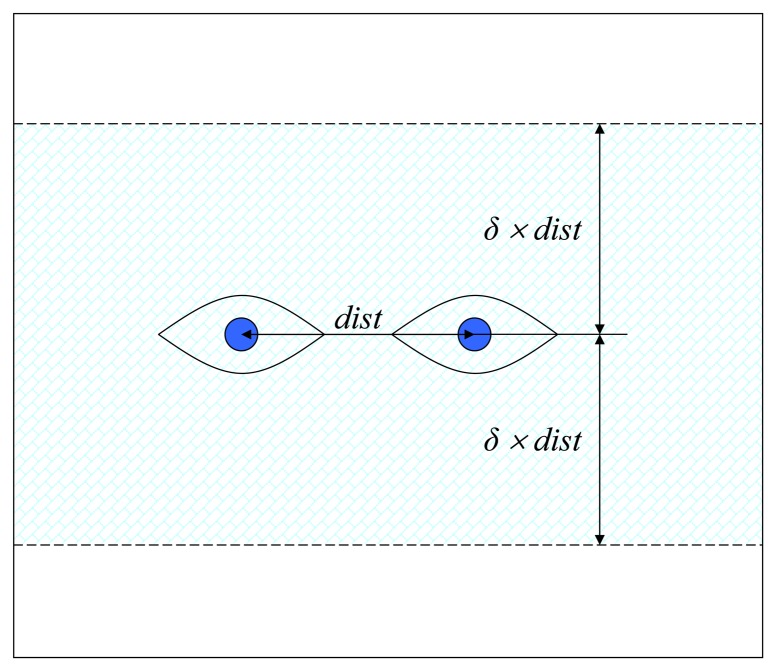
Eyeglasses search area. The eyeglasses search space is fixed to a region surrounding the eyes.

The interpupillary distance (IPD) is approximated as the distance between the center of the rectangles corresponding to each eye. The information regarding the interpupillary distance is used to delimit the region in which the eyeglasses are likely to be found, in a similar manner as presented in [3]. Figure 6 depicts the computed search area of the eyeglasses based on the eyes position and the approximation of the interpupillary distance.

We experimentally determined that *δ* = 1.5 provides enough accuracy to detect the eyeglasses ROI for most of the human faces. Increasing the value of *δ* would unnecessarily search for solutions too far away from the eyes, while decreasing it would fail to encompass the entire contour of bigger-sized eyeglasses.

Alternate methods could be used for both detecting the eyes ([16]) and establishing the eyeglasses search area. For example, the eyeglasses region of interest could be robustly extracted using *Active Appearance Models* ([24]) or *Constrained Local Models* ([25]). We chose, however, to compute this zone heuristically, as presented in [3,5], because it is very simple, does not require a training phase and complies with the conditions we imposed for the hypotheses generation space. This region must be large enough to comprise the contours of the eyeglasses and sufficiently narrow, such as the search space to be limited. Of course, the method of computing the search region of the eyeglasses and the method of localizing the eyes can be replaced at any time, as the eyeglasses detection algorithm (the main contribution of this work) takes as input the eyeglasses search area.

### 4.5. Monte Carlo Sampling

Monte Carlo Methods ([26]) refer to a broad class of algorithms that solve a problem by generating random samples from a probability distribution over the input domain and by observing the fraction of the samples obeying several properties. These algorithms are often used for obtaining numerical solutions to problems that are too complex to be solved analytically or are infeasible for deterministic algorithms.

Eyeglasses rims have different shapes, and an analytical description of their contour is impractical. Moreover, the eyeglasses region is often occluded by several other elements, and it is very hard to distinguish between the pixels that belong to the eyeglasses lenses and those belonging to other elements.

To overcome these problems, we apply a Monte Carlo method to determine the position of the eyeglasses and the contour of the lenses, by randomly generating a large number of hypotheses and clustering them based on their position and size, as shown in Algoritm 1.



**Algorithm 1:** Monte Carlo sampling.
 **Input:** eyes position, interpupillary distance  Define the possible domain of inputs for each dimension of the hypothesis.  **while***number*_*of*_*generated*_*hypotheses* ≤ *max*_*hypotheses***do**   Generate random hypothesis by sampling from a uniform probability distribution over the domain.   Match the hypothesis over the input image.   *number*_*of*_*generated*_*hypotheses* ← *number*_*of*_*generated*_*hypotheses* + 1  **end while**  Cluster the hypotheses based on their position and size.  Select the best cluster based on its matching score.


**Figure 7.**
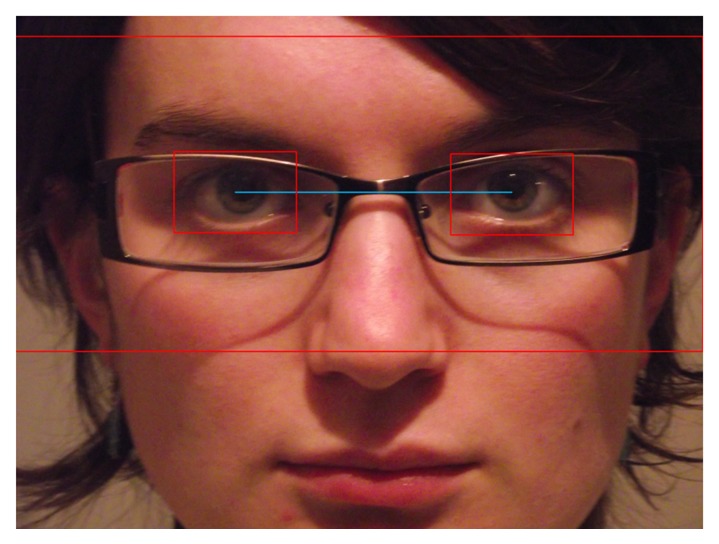
Eyeglasses region of interest (ROI). The detected position of the eyes are marked with red rectangles. The approximated interpupillary distance is marked with a blue line, and the eyeglasses search area is delimited by a red rectangle.

Prior to this sampling procedure, the eyeglasses search space is restricted to an area that surrounds the eyes (as described in Section 4.4). Figure 7 depicts the eyeglasses search area; all the hypotheses will be generated within this region.

As stated in Equation (4), a hypothetical rim is defined by the following parameters: the position (the centroid of each one of the eyeglasses lenses), the size of the lenses and the *Fourier coefficients* that describe the contour of the lenses. For the construction of a new hypothesis, two reference shapes are selected from the database; next, the position, the size and the interpolation factor between the reference shapes are drawn from a uniform probability distribution.

The centroids of the eyeglasses lenses are obtained by sampling points from a region determined by the position of the eyes and the interpupillary distance. The size of the lenses is drawn from an interval determined by the interpupillary distance. To determine the shape of the new hypothesis, we choose two shapes from the database and morph them with an interpolation factor within [0, 1], in order to obtain the *Fourier coefficients* that describe the contours of the lenses.

Each hypothesis is validated and assigned a non-negative score by matching it over the *Distance transform* image (Section 4.2).

As stated in Section 4.3, the eyeglasses rims are classified into three distinct classes: rectangular symmetrical rims, elliptic symmetrical rims and asymmetrical rims. The same Monte Carlo sampling procedure is performed for each one of these classes. At the end of the sampling procedure, all the valid hypothesis are grouped into clusters, depending on their position and size, as shown in Algorithm 2. We used an *agglomerative clustering approach*: initially, all data points belong to a separate cluster, and then, clusters are successively merged based on their similarity.



**Algorithm 2:** Hypotheses clustering algorithm.
 **Input:** sequence of hypothesis *H* **Output:** clustering of hypotheses based on position and size: *C*  *C* ← *H*  **while** clusters can be created **do**   pick *c_i_*, *c_j_* ∈ *H* the two nearest clusters   *c_k_*← cluster (*c_i_*, *c_j_*)   *C* ← *C* — {*c_i_*, *c_j_*}   *C* ← *C* ∪ { c*_k_*}  **end while**


The standard hierarchical clustering algorithm terminates when all the clusters have been merged into a single cluster or after a predefined number of steps.

Two clusters are susceptible candidates for merging if the overlapping area of the bounding rectangles of each cluster is at least *α*%. Based on this criterion, the clustering procedure ends when there are no more clusters that can be grouped into a new one. In our experiments, we used *α* = 90% in order to ensure the compactness of the clusters.

The determined clusters provide information regarding the regions where the eyeglasses are likely to be located. Clusters with good matching scores are more likely to contain the eyeglasses. This clustering process eliminates false positives: for a false positive, even if it has a good matching score, the other hypotheses from its cluster are more likely to have a bad score; while in the cluster that contains the most similar shape to the eyeglasses all shapes have a good matching score.

**Figure 8.**
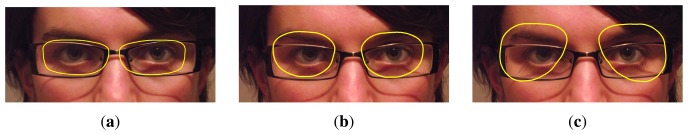
Intermediate result after Monte Carlo and clustering. (**a**) Rectangular rims; (**b**) elliptical rims; (**c**) asymmetrical rims.

The hypothesis used in the next step of the algorithm is the best hypothesis from the cluster with the best matching score. At the end of this step, we determined the approximate position and size of the eyeglasses rims. The result of the Monte Carlo search is illustrated in Figure 8 for each one of the three eyeglasses classes.

### 4.6. Shape Adjustment

After the Monte Carlo search, the approximate location, size and shape of the eyeglasses rims are determined.

The problem now is to finely tune these parameters in order to obtain a solution as close as possible to the actual eyeglasses in the image. To address this problem, we perform a random walk around the solution found in the Monte Carlo search, as shown in Algorithm 3.



**Algorithm 3:** Shape adjustment algorithm.
 **Input:**
*x*_0_ initial solution after Monte Carlo search, *max*_*iterations* number of iterations **Output:**
*x'* final solution of the algorithm  *x* ← *x*_0_  *iteration* ← 0  **while**
*iteration* < *max*_*iterations*
**do**   generate *x'* by sampling from the distribution *P*(*x'*∣*x*)   evaluate *x'*   **if**
*score*(*x*) < *score*(*x'*) **then**    (*x*) ← *x'*   **end if**   *iteration* ← *iteration* + 1  **end while**


At each step of the algorithm, a new sample, *x'*, is generated by randomly picking a value from the normal probability distribution centered in the current solution, *x*: *P*(*x'*∣*x*). *P*(*x'*∣*x*) is used as a motion model in the state space and suggests a candidate for the next sample, *x'*, given the previous sample, *x*. We chose to use the Gaussian distribution centered at *x*, so that the points closer to *x* are more likely to be visited next. If the new sample, *x'*, is more likely than the previous one, *x*, the current solution is set to *x'*. The generated samples are evaluated by matching them over the *Distance transform* image and validated as presented in Section 4.2. To generate the new candidate, *x'*, we change only one of the dimensions (i.e., the size, the position or the shape) of the current sample, *x*, by drawing from the Gaussian distribution, *P*(*x'*∣*x*).

The algorithm is run in an iterative way for *max_iterations* = 30. The value for the maximum number of iteration was determined heuristically, after multiple tests. We observed that increasing the maximum number of iterations over 30 has little to no impact in the majority of the cases.

**Figure 9.**
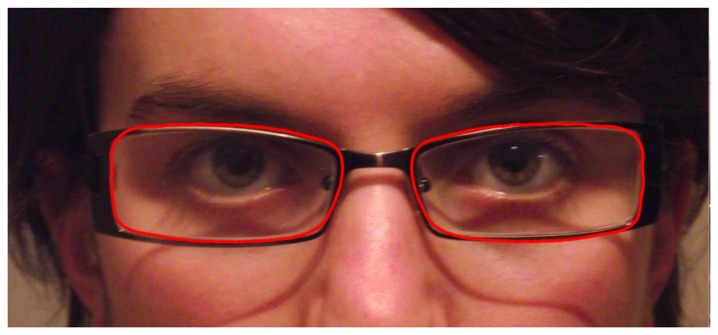
Final result of the algorithm. The extracted lenses contours are marked in red in the image.

The algorithm is run for each one of the classes (elliptic, rectangular and asymmetrical eyeglasses rims). The solution with the best matching score is selected as the final result. An example of the eyeglasses detection algorithm is depicted in Figure 9.

## 5. Tests and Results

The eyeglasses contours extraction algorithm is targeted for optometric measurement systems: we plan to determine the exact shape of the rims and to accurately measure several morphological parameters ([27]): the boxing size, the vertex distance and the wrap angle.

Part of this research grant is supported by one of the major optometric measurement systems manufacturers ([4]).

**Figure 10.**
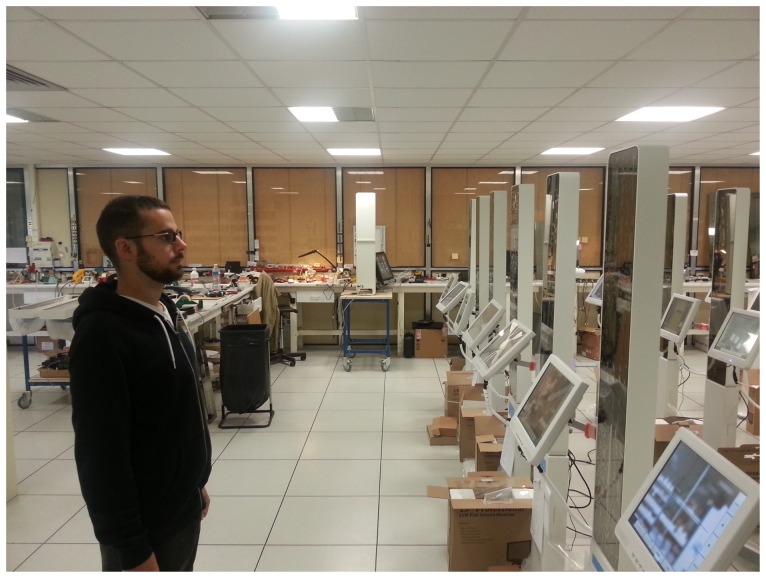
Experimental setup: the patient stands still in front of the device with his head in a vertical position.

The measuring devices produced by the company determine the morphological parameters needed by any eyeglasses prescription by processing a digital image of the patient. For the measurement process, the patient (wearing eyeglasses) stands still in front of the device at a distance varying from 1 to 2 m, and the device captures an image of the patient's face using a high resolution camera, as shown in Figure 10. The morphological parameters are determined by detecting the interest points (the center of the pupils, the frames of the eyeglasses) on this image and computing the relationship between them in real world coordinates (mm or degrees). All these devices have been validated by comparing the result of the measurements obtained using this computer-aided method and those obtained using the classical methods from the optometry field. For the validation process, several optician stores participated in a trial study and provided the company images of patients captured in real life conditions. Currently, for the extraction of the eyeglasses lenses, these devices use an algorithm that provides only an approximation of the rim shape. The optician must manually adjust the contour of the eyeglasses for each image.

The dataset we used for testing our algorithm is a subset of the database used in the validation process, over 300 facial images of people wearing different types of glasses. An XMLfile containing the position of the pupils, the points of the contour of the eyeglasses (manually marked by the optician), the measurements computed by the machine and the measurements performed by the optician is attached to each image from the database. We used a subset of this database to test the eyeglasses lens contour extraction algorithm.

As our solution is targeting this type of measuring devices, our testing database contains no facial images without eyeglasses.

The metric we used for the testing procedure of the eyeglasses extraction algorithm is the distance between the detected eyeglasses rim and the actual rim from the input image. An outcome of the algorithm is considered a true positive if the overlapping area between the extracted contour and the real contour of the lens from the input image is larger or equal to *γ* = 0.95 of the actual eyeglasses rims and a false positive otherwise. The detection rates of our algorithm are shown in Table 2.

The eye region was not detected in images where the lenses of the eyeglasses contained multiple large reflections.

These results demonstrate that our method works for a variety of samples with different face profiles, eyeglasses shapes and illumination.

**Figure 11.**
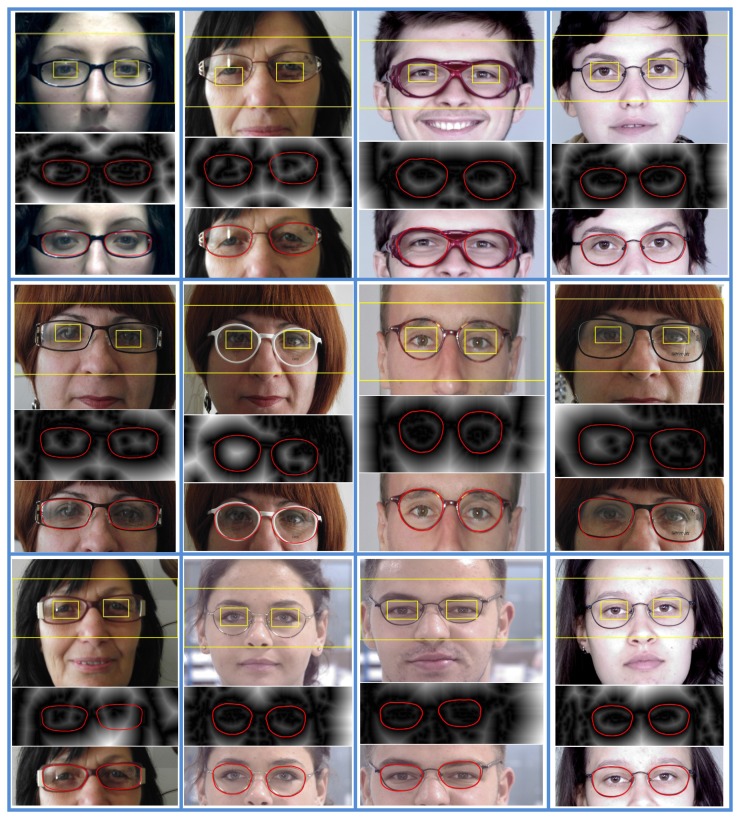
Lens contour extraction results.

Eyeglasses rim extraction results are shown in Figure 11; one can notice, on each vertical group of images, the region of the eyes, as well as the search area of the eyeglasses and the extracted eyeglasses lens contours on the distance transform image and on the original image, respectively.

The execution time of our eyes and eyeglasses detection algorithm on a regular PC (Intel Core 2 CPU T7200, 2.00 GHz, 2 GB memory) is 1.1 s for images with a resolution of 1,600 × 1,200 pixels.

**Figure 12.**
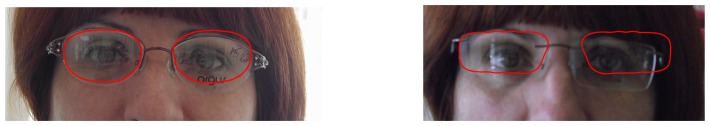
Eyeglasses contour extraction—some failure cases. The lens contours are not accurately extracted from images with little edge information.

The detection process is based only on edge information, as the generated hypotheses are matched against the *Distance transformation* of the Canny edge image; therefore, the algorithm does not yield accurate results on images with inadequate edge information, such as is the case for rimless eyeglasses. Figure 12 presents some failure cases:

## 6. Discussion

In this section, we perform a comparative study between the previous methods proposed in the literature and our solution for extracting the eyeglasses.

Much of the work presented in the literature is based on eyeglasses removal from facial images ([7,8,12,14]), and other articles are focused on eyeglasses detection to decide whether the eyeglasses are present or not in the input image ([3,15]).

The scope of this paper is to precisely extract the contour of the eyeglasses lenses, and not to remove glasses or to determine their presence in the input images. Given this goal difference, we could not establish any relevant comparison criterion between these operations on eyeglasses. Even for the previous eyeglasses contour extraction methods, the metrics used for deciding the true positive and false positive cases differ from one work to another or are even not specified.

Moreover, the testing databases used for the validation of these algorithms are not publicly accessible, standard datasets, and therefore, we could not test our implementation on the same images to compare the algorithms' performances.

Table 3 illustrates the comparison between the state-of-the-art papers on eyeglasses detection, contour extraction and removal and our method, as well as the performance rate of these algorithms, even though they have been tested on different datasets.

Based on this comparison, we can conclude that the proposed algorithm for eyeglasses contour extraction yields very good results, which are at least comparable with the ones obtained in the previous reported implementations, and in some cases, even better.

The eyeglasses detection algorithm is targeted for optometric measurement systems, such as [1,2,4]. Therefore, we did not intend to decide whether the eyeglasses are present or not in the image, but to extract an as precise as possible contour of the eyeglasses lenses. Our algorithm assumes that the eyeglasses are present in the input image.

In such systems, in order to perform an accurate measurement, the patient should stay as still as possible, with the head in a vertical position. Therefore, we did not necessarily focus on facial pose variation: the algorithm is designed to work on frontal facial images taken under these conditions and not for profile face images or for images under extreme face variations.

However, we took into consideration the heads horizontal tilt angle, as most persons' natural standing pose implies a certain (horizontal) tilt of the head. Both the eye detection algorithm and the eyeglasses detection algorithm perform well if the head horizontal tilt angle is less than 10 degrees: when searching for the eyes and the eyeglasses, we imposed a condition on the maximum slope between the left and the right eye/eyeglasses lens.

If the head horizontal tilt angle is bigger than 10 degrees, the image is no longer useful for the measurement process, because it induces aberrations for which they can no longer be compensated.

## 7. Conclusions and Future Work

This paper presents a system that automatically detects the position and the shape of eyeglasses in facial images. The system consists of three modules: eyes localization, eyeglasses localization and eyeglasses shape refinement and is based on hypothesis testing. The first module is used to restrict the search area of the eyeglasses. After the search area of the eyeglasses was established, we used a Monte Carlo search to detect the approximate position and shape of the lenses. The search space of the eyeglasses rims is explored using a database that contains representative shapes of the lenses. In the final step of the algorithm, we performed a random walk around the approximate solution to finely tune the position and the contour of the eyeglasses rims.

We proposed an original model for representing the lenses based on *Fourier descriptors* that has several advantages. First of all, a large number of eyeglasses shapes can be easily represented by using only a few variables (we used 14 coefficients to describe the eyeglasses). Secondly, we proposed an original strategy for morphing between the shapes based on these descriptors. This morphing method allows us to generate new hypotheses and, thus, to explore the search space, using a finite number of shapes stored in the eyeglasses database. Moreover, the *Fourier descriptors* are invariant to affine transformations, such as scaling, translation and rotation.

Our approach is not limited to the detection of eyeglasses, and it can be applied to the detection of a variety of visual objects. For this, we only need to create a database of representative shapes of the objects we want to detect.

We performed multiple experiments to demonstrate the effectiveness of the proposed solution. A recognition rate of 92.3% and the localization results shown in Figure 11 demonstrate the effectiveness of our system.

As future work, we plan to include additional information, besides the edge information we currently use, for our matching criterion in order to improve the segmentation quality of our solution.

## Figures and Tables

**Table 1.** Eyeglasses database samples example. The table presents a sample for each one of the eyeglasses classes, along with the corresponding *Fourier descriptors*.

**Class**	**Eyeglasses Rim Contour**	**Fourier Descriptors**
Symmetrical rectangular rims	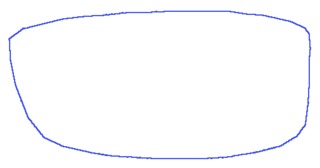	(4430, −454), (−57.3, −379), (1,110, −40.5), (−151, −416), (37.1, 303), (−88.4, −176), (−352, 255), (−566, −283), (−119, 281), (710, −375), (−29.4, 483), (5,040, 35.8), (334, 258), (24,700, 605)
Elliptical symmetrical rims	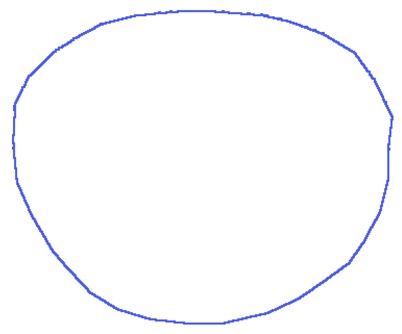	(874, 479), (185, −162), (684, 292), (18.5, 60.8), (185, 156), (−121, −223), (−28.6, −211), (−381, 214), (−119, 339), (−638, 308), (−12.7, 549), (1,650, 168), (285, −271), (23,200, -396)
Asymmetrical rims	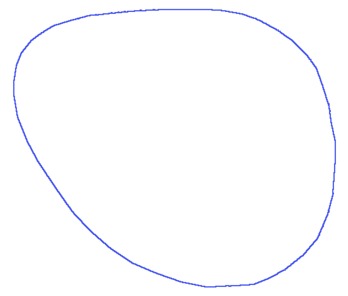	(1750, −119), (−68.3, −526), (403, 136), (−142, −102), (217, 96.8), (−94.4, 2.60), (26.7, −71.9), (−554, −226), (−275, 203), (−289,−189), (−276, 358), (3,000, 77.4), (377, 108), (25,400, 813)

**Table 2.** Detection rates.

**Detection Module**	**Detected**	**False Negatives**	**False Positives**
Eye detection	98%	2%	0%
Lenses contour extraction	92.3%	2.5%	5.2%

**Table 3.** Comparison of the proposed method with related works.

**Work**	**Operation on eyeglasses**	**Method**	**Dataset size**	**Performance**
Jiang *et al.* [3]	Detection	Edge information Geometry	80	Metric: Fisher criterion (J); can not compare

Jing *et al.* [5]	Detection Extraction	Deformable contours	419	Extraction rates: Accurate results: 50%; Satisfactory results: 80%

Jing *et al.* [6]	Detection Extraction	Bayes rule	100	Mean average error (pixels): Left lens: 11.09; Right lens: 19.62

Wu *et al.* [11]	Extraction	3D Hough Transform	513	Frames only: 80%; Frames + eyelids/irises: 90%

Park *et al.* [7]	Removal	Recursive error compensation PCA reconstruction	100	Metric: difference between input image and image without glasses; can not compare

Park *et al.* [8]	Removal	Recursive PCA reconstruction	264	Metric: Euclidian distance between input image and reconstructed image; can not compare

Wu *et al.* [12]	Eyeglasses localization Removal	MCMC Statistical analysis	264	Localization: 15 keypoints on the eyeglasses 95%

Proposed solution	Extraction Shape recognition	Fourier descriptors, Monte Carlo	363	92.3%
